# Location Matters: How Spatial Positioning of Regulatory Proteins Produces Divergent Outcomes

**DOI:** 10.1371/journal.pbio.1000333

**Published:** 2010-03-16

**Authors:** Richard Robinson

**Affiliations:** Freelance Science Writer, Sherborn, Massachusetts, United States of America

**Figure:**
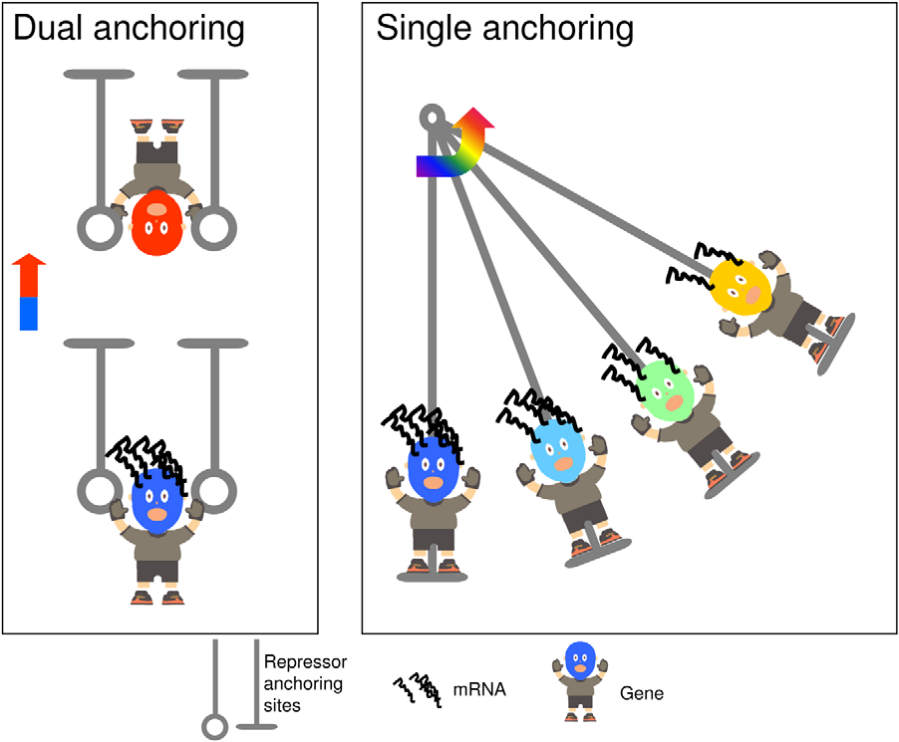
A gene stretched between two repressor anchoring sites can assume two stable positions. Conversely, single anchoring lifts the gene gradually.

“What turns a gene on?” may sound like the set-up to a sophomoric biology joke, but in fact it is a central question in molecular biology, crucial to understanding how cells survive and develop. The broad outlines—transcription increases when the local chromosomal environment is opened up and decreases when it condenses—and even many of the details are well known. On the other hand, a quantitative understanding of the regulatory logic governing the activation or repression of any particular gene is less well known. In a new study, Janos Kelemen, Attila Becskei, and colleagues elucidate one aspect of that logic, showing that the same regulatory protein can have two quite different effects, depending on the number and spatial positioning of its binding sites around its target gene.

The regulatory regions of a eukaryotic gene are complex and include both enhancer and repressor binding sites. Enhancer sites bind activator proteins, which increase the activity of the transcription machinery. Repressor sites bind silencing proteins, which decrease transcription, either by interfering with the machinery directly, or by linking up with one another, forming inhibitory chromosomal structures that prevent access to the gene within.

To explore the effect of silencer positioning, the authors flanked a fluorescent reporter gene in yeast with binding sites for the silencing protein Sir3p. They then added both silencer and activator proteins, and watched what happened. They found that with an intermediate level of activator, some cells lit up, whereas others didn't—the gene was essentially switched all the way on in some, but all the way off in others.

The ability of a system to exhibit two stable states—on and off—under one set of conditions is called bistability. To understand how bistability could arise in their system, the authors constructed a mathematical model. It took the form of a “reaction-diffusion” equation, with each term representing one influence on the concentration of the silencing protein, including its ability to slide along the DNA until it finds its binding site, its propensity to associate with other Sir3p molecules, and the mutually reinforcing effects of chromatin condensation on protein concentration. Importantly, when Sir3p binds to the chromosome, it acts as a “nucleation site” for other Sir3p proteins, a process that speeds up as more proteins bind.

By running simulations with their model, they showed that when there were two silencing sites on opposite sides of the gene, two different patterns of Sir3p concentration over time could arise, depending on the initial concentration of the protein. When the initial concentration was high, the two sites reinforced one another, with nucleation at one speeding up the accumulation of protein at the other. But when the initial concentration was low, the effect of the two silencing sites remained isolated, with a weak accumulation at both sites.

When the authors varied activator levels in the simulations, they reproduced their experimental observations. At intermediate levels, bistability emerged, predicting that some cells in a population—those with high initial Sir3p—remain “off,” whereas others—those low in Sir3p—switch “on.”

The bistable pattern collapsed, however, when they removed one of the binding sites. In that situation, there was only a single source for Sir3p nucleation and no reinforcement between sites. Instead, the system exhibited “monostability”—cells exhibited a graded, rather than on/off response to increasing levels of activator. Further tinkering with the model indicated that weakening the strengths of interaction among Sir3p proteins could turn a bistable system into a monostable one, as well.

The quantitative understanding of how the spatial pattern of silencer binding sites distinguishes bistability from monostability is likely to have some important consequences. In silico, it should allow researchers to fine-tune model systems to better approximate gene expression in living systems. It should also lead to a better understanding of the more complex regulatory control underlying expression of individual genes in vivo. Although the experiments in this study were performed in yeast, it is likely that the findings are relevant to humans as well, given that at the level of gene regulation eukaryotes of all kinds share many of the same fundamental mechanisms.

**Kelemen JZ, Ratna P, Scherrer S, Becskei A (2010) Spatial Epigenetic Control of Mono- and Bistable Gene Expression. doi: 10.1371/journal.pbio.1000332**

